# Evaluating the Individualized Treatment of Traditional Chinese Medicine: A Pilot Study of N-of-1 Trials

**DOI:** 10.1155/2014/148730

**Published:** 2014-11-11

**Authors:** Haiyin Huang, Peilan Yang, Jingjing Xue, Jie Tang, Liyu Ding, Ying Ma, Jie Wang, Gordon H. Guyatt, Thuva Vanniyasingam, Yuqing Zhang

**Affiliations:** ^1^Department of Respiratory Disease, Yueyang Hospital of Integrated Traditional Chinese and Western Medicine, Shanghai University of Traditional Chinese Medicine, Shanghai 200437, China; ^2^Department of Pharmacy, Yueyang Hospital of Integrated Traditional Chinese and Western Medicine, Shanghai University of Traditional Chinese Medicine, Shanghai 200437, China; ^3^Department of Clinical Epidemiology and Biostatistics, McMaster University, Hamilton, ON, Canada L8S 4K1

## Abstract

*Purpose*. To compare the efficacy of individualized herbal decoction with controlled decoction for individual patients with stable bronchiectasis. *Methods*. We conducted N-of-1 RCTs (single-patient, double-blind, randomized, multiple crossover design) in 3 patients with stable bronchiectasis. The primary outcome was patient self-rated symptom scores on visual analogue scales. Secondary outcome was 24-hour sputum volume. A clinical efficacy criterion which combined symptoms score and medication preference was also formulated. *Results*. All three patients showed various degrees of improvement on their symptoms and one patient's (Case 3) 24 h sputum volume decreased from 70 mL to 30 mL. However, no significant differences were found between individualized herbal decoction and control decoction on symptoms score, or on 24-hour sputum volume. One patient (Case 2) had clear preference for the individualized herbal decoction over the standard one with the confirmation after unblinding. We therefore considered this case as clinically important. *Discussion*. N-of-1 trials comply with individualized philosophy of TCM clinical practice and had good compliance. It is necessary to set up clinical efficacy criteria and to consider the interference of acute exacerbation.

## 1. Introduction

Single case randomized controlled trials (N-of-1 randomized controlled trials, referred to as N-of-1 RCTs or N-of-1 trials) have been paid more and more attention since clinicians started realizing the limitation of population-based randomized controlled trials (RCTs) when medical interventions work ubiquitously (or under most circumstances) for the majority of common chronic conditions [1–3]. It is well known that treatment based on syndrome differentiation is one of the characteristics and essences of traditional Chinese medicine (TCM) which emphasizes individualized treatment [4, 5]. This individualized TCM intervention often makes it difficult for population-based RCTs to carry out a standard form. The use of decoctions with fixed herbs or patent Chinese medicine hardly represents the superiority of individualized treatment effects. Thus population-based RCTs might not be the optimal study design for TCM in nature [4, 5]. N-of-1 trial, on the other hand, ensures the comparison between classical syndrome differentiation treatment and non-syndrome differentiation treatment with rigorous methodological design [4]. It may be an adequate study design for TCM since it well represented the individualized treatment philosophy of TCM.

Bronchiectasis is a chronic condition defined by permanent dilation of the bronchi. The cause could be either idiopathic or associated with other diseases (states). The incidence of bronchiectasis in a given community is largely unknown which varies from 3.7/100000 to 52/100000 in New Zealand and in the USA [6]. Bronchiectasis is one of the common chronic respiratory diseases in China [7]. Treatment regimens are not well defined and remain largely empirical, and patients tend to have ongoing symptoms and lung function decline despite the management. Although antibiotic therapy has been used commonly, its use in the stable stage of bronchiectasis is still controversial [6, 7]. TCM plays an important role in the management of bronchiectasis in China. There is no standard traditional Chinese herbal decoction for stable bronchiectasis; the principles of treatment are based on TCM syndrome differentiation including reducing phlegm, clearing the lung heat, and strengthening healthy energy [8]. Some studies found that heat-clearing drugs such as* Scutellaria baicalensis* and Radix Arnebiae seu Lithospermi have antibacterial activities against* Pseudomonas aeruginosa* and other bacteria [9, 10]; Radix Astragali and* Poria cocos* can strengthen the function of immune system;* Platycodon grandiflorum* and Rhizoma Fagopyri Cymosi have strong sputum-removing effect [11]. We found in a previous randomized controlled trial that syndrome differentiation treatment based on the bronchiectasis stabilization decoction can improve the clinical symptoms, reduce the annual frequency of acute exacerbations, improve the quality of life, and delay FEV1 (forced expiratory volume in one second) decline of the patients in stable stage of bronchiectasis, with no severe adverse effect [8].

Considering the special challenges of conducting N-of-1 trial in TCM (e.g., the unclear half-life period of TCM decoctions), we conducted this pilot study comparing the efficacy of individualized herbal decoction with control decoction (bronchiectasis stabilization decoction) in patients with stable bronchiectasis to assess its feasibility.

## 2. Methods

### 2.1. Study Design

**Figure 1:**
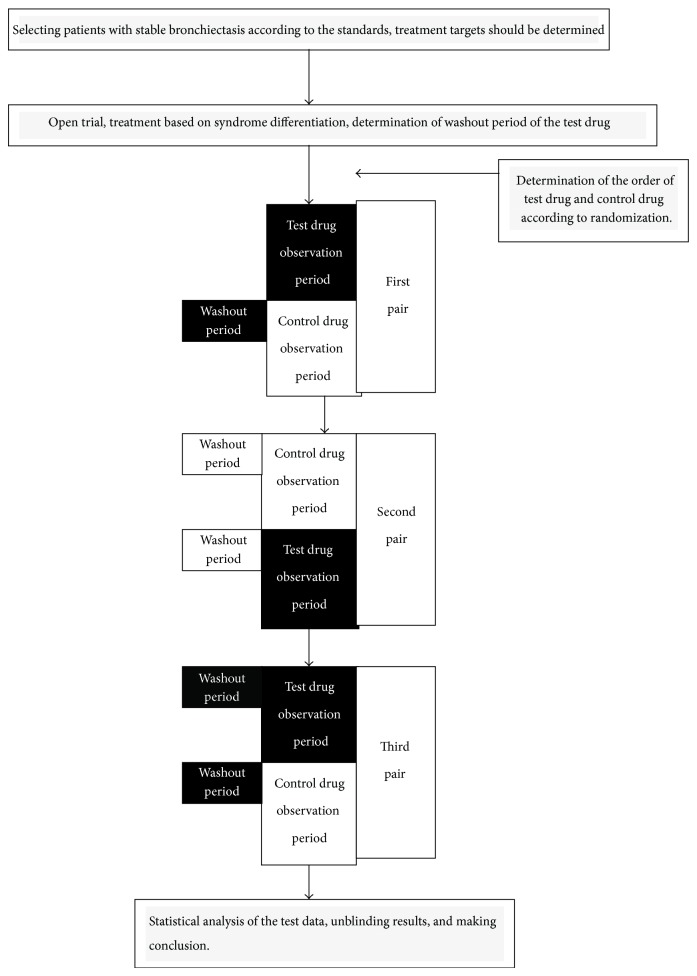
The flow chart of the N-of-1 trials in the treatment of stable bronchiectasis by traditional Chinese medicine based on syndrome differentiation.

These N-of-1 trials were randomized, double-blind, crossover comparisons of individualized herbal decoction with control decoction within individual patients. N-of-1 trials were offered to the patients meeting the inclusion criteria and who had shown a clinical response to TCM in an open preliminary trial. With changes in patients' self-rated symptom scores as the main outcomes, preliminary trials can obtain onset time after drug administration and efficacy maintenance time after drug withdrawal, so as to determine the observation period (2-3 weeks) and the washout period. Each N-of-1 trial lasted 12–18 weeks and consisted of three cycles with treatment and control assigned in random order. We measured outcomes in the last week of each observation period, and the time (1-2  weeks) before this was supposed to be the washout period (Figure 1). If acute exacerbation of bronchiectasis occurred, antibiotics and other treatments were provided conventionally [6, 7]. We resumed the study when infection was controlled and the disease returned to stable stage.

As herbal decoction is a mixture of herbs, it is difficult to determine the half-life period biochemically. We decided to conduct the preliminary study due to the unique characteristics of TCM decoction.

### 2.2. The Process of the Preliminary Trial

The definitions of some evaluation criteria are as follows.

(*1) The Baseline for Patient Self-Rated Symptom Score.* Since all the patients were in stable condition of bronchiectasis, the baseline of patient self-rated symptom score on visual analogue scale (VAS) was set as the average value of symptom scores on VAS in three consecutive days before the trial. The mean of baseline scores for symptoms was defined as the value of baseline divided by the kinds of symptoms (such as cough, expectoration, shortness of breath, chest pain, fatigue, etc.).

*(2) The Onset Time Point*. The first day is when the mean symptom score is reduced by 0.5 or more than the mean of baseline after drug administration, and the result is maintained in three consecutive days, without relapse.

*(3) The Efficacy Maintenance Time Point*. The first day is when the mean symptom score increases by 0.5 or more than the mean of baseline after drug withdrawal, and the result is maintained in three consecutive days, without reversal.

*(4) Washout Period*. It is the time from drug withdrawal to the efficacy maintenance time point.

*(5) Observation Period*. The observation period must be longer than the onset time point and must be at least one week longer than the washout period.

The open-label preliminary trial was carried out according to the above evaluation criteria. With changes in patients self-rated symptom scores as the main outcomes, preliminary trials can obtain onset time after drug administration and efficacy maintenance time after drug withdrawal, so as to determine the observation period and the washout period.

### 2.3. Patients and Diagnosis

Outpatients were eligible if they meet the following criteria: (1) the diagnostic criteria based on the consensus of Chinese experts [7] and the guidelines for noncystic fibrosis bronchiectasis issued by the British Thoracic Society in 2010 [6]; (2) male or female, aged 18–75 years; (3) being in the stable stage, and no acute exacerbation of bronchiectasis within the past three weeks; (4) frequency of acute exacerbation of bronchiectasis ≤ 3 times every year; (5) signed informed consent for participation. The exclusion criteria include (1) having developed respiratory failure with estimated survival time less than one year; (2) having hemoptysis as a comorbidity; (3) having complications by active tuberculosis; (4) being pregnant or with severe heart, liver, and kidney dysfunctions; (5) participating in other pharmacological clinical trials within the past 3 months.

TCM syndrome diagnostic criteria were based on the “TCM standards for diagnosis and efficacy of diseases” issued by the State Administration of Traditional Chinese Medicine [12] and integrated with the TCM differentiation of bronchiectasis summarized from the literature [13]: mainly including lung and spleen deficiency syndrome, qi and yin deficiency syndrome, and phlegm-heat obstructing lung syndrome (including mild phlegm-heat syndrome). Patients with corresponding two primary symptoms or more than two accompanied symptoms with the corresponding tongue and pulse signs could be diagnosed as having the TCM syndrome.

To ensure the quality and accuracy of TCM syndrome differentiation, TCM syndrome of each patient should be independently assessed by two associate chief physicians (or higher-title physicians). If there is any controversy, it should be decided by a third party (distinguished veteran doctor of TCM).

### 2.4. Randomization and Blinding

We used block randomization and the block size was 2. The orders in which patients receive drugs were randomized by computer for each single case, such as BA-AB-BA or AB-BA-BA. Doctors prescribed both individualized prescription and control prescription after assessing patients' TCM syndrome. Then the two prescriptions together with the randomized medication order were delivered to a pharmacist specifically designated by the TCM Pharmacy. The pharmacist used the coin method to determine which one of A or B represented individualized prescription or control prescription, recorded the blind code, and put it for safe keeping. Then the pharmacist prepared the herbs of the prescription following the randomized medication order. The decoction of TCM was made in the decoction room of our hospital and dispensed to the patient. This method successfully kept the doctor blinded during the contact between doctors and the pharmacist. The test drug and control drug had no differences in dosage form, appearance, color, specification, label, and so forth. Doctors, patients, and outcome assessors were all blinded.

### 2.5. Interventions

Patients took Mucosolvan 60 mg three times daily to reduce phlegm in both intervention and control period and chest physical therapy, mainly, including postural drainage and chest percussion to help expel the sputum. If acute exacerbation of bronchiectasis occurred, antibiotics and other treatments were provided [6, 7].

Concomitant treatments were used at the same time for other chronic diseases such as hypertension, coronary heart disease, and diabetes, but the usage should be relatively fixed. Detailed medication records should be made.

*(A) Bronchiectasis Stabilization Decoction (Control Decoction, CD) Applied in the Control Drug Observation Period*. This decoction (CD) contains eight herbs: Radix Lithospermi 15 g, Rhizoma Fagopyri Cymosi 30 g, Radix Ophiopogonis 15 g,* Poria cocos *15 g, Radix Astragali 20 g, Rhizoma Bletillae 10 g,* Platycodon grandiflorum* 10 g, and Semen Coicis 30 g. 

*(B) Syndrome Differentiation Decoction (Individualized Decoction, ID) Applied in the Tested Drug Observation Period*. ID was the modification of CD based on syndrome differentiation. For example, for patients with lung and spleen qi deficiency syndrome, we added* Codonopsis pilosula*, Pericarpium Citri Reticulatae, and Rhizoma Atractylodis Macrocephalae; for patients with qi and yin deficiency syndrome, we added Radix Adenophorae, Radix Glehniae, and Radix Rehmanniae Recens; for patients with obvious phlegm-heat syndrome, we added* Scutellaria baicalensis* and Herba Violae. Besides, the herbs in a prescription could be changed according to different symptoms of individual patients.

Pieces of TCM which had passed quality inspection in line with the national norms were provided by the hospital pharmacy. The decoction of TCM was made according to the literature [14] in the decoction room of our hospital. Pieces of TCM were wrapped in nonwoven cloth bag, soaked in water for 30 min, and decocted 1 time for 60 min in a TCM decocting machine manufactured by Tianjin Sanyan Precision Machinery Ltd. (model: DJQ252). The Chinese herbal decoctions were taken by one decoction a day and divided into 2 doses.

### 2.6. Outcome Measures

The referring physician saw the patient before and after each treatment period and collected data. We asked the patient to identify the symptoms that bother them and a self-administered patient diary or questionnaire was made. The following were three outcome measures.

#### 2.6.1. Primary Outcome: Patient Self-Rated Symptom Score

Patients rated the severity of the symptoms (such as cough, expectoration, shortness of breath, chest pain, and fatigue) on visual analogue scales. The higher the score, the more severe the symptom [1, 15]. Taking cough as an example, on average, in comparison with your usual cough, how severe was the cough?

1. No cough, or as mild as, or milder than they have ever been.
2. Not nearly as severe as usual.
3. Not as severe as usual.
4. As severe as usual.
5. Severer than usual.
6. Very severe, almost as severe as they have ever been.
7. Very severe, as severe as or more severe than they have ever been.

We consider an improvement of 0.5 points per question corresponds to a noticeable improvement in the patient's well-being. If there were seven questions, a total change of 3.5 or more points was considered clinically important [1, 15].

#### 2.6.2. Secondary Outcomes

*(1) 24 h Sputum Volume*. We measured the 24 h sputum volume and took the mean value for the 3 consecutive days at the beginning and the end of each trial. To ensure the accuracy of the measurement, we asked the patients to spit sputum into a collector with scales from 8:00 am to the next 8:00 am. We used the mean value of the sputum volume for 3 consecutive days as the outcome.

*(2) Safety Outcome*. We recorded adverse events which occurred and, if necessary, terminated the trial and unblinded the code.

*(3) Feasibility Outcome*. According to the literature [16], we made the criteria for determining success of the pilot study: (a) recruitment rate: at least 50% of all eligible patients can be recruited, (b) completion rate: at least 70% of all recruited subjects complete the study, (c) at least 90% of patients had to receive every scheduled prescription of the study drug in a blinded manner, and (d) the clinical outcomes are easily obtained.

### 2.7. Data Analysis

According to the literatures [1, 17], we made the following standards of clinical efficacy criteria.

We considered the effect as clinically significant if the difference in the mean symptom score of at least two pairs out of three pairs is more than 0.5 points or both the clinician and patient are convinced that the experimental therapy is effective.

The values of the outcomes were measured after the washout period after each pair which started in the last week of each observation period, to avoid the carry-over effects of the previously used drug. We took the mean value of the data collected from the last week of each observation period and then conducted statistical analyses [15].

All statistical analyses were performed using RStudio 0.98.953. One-sided paired Wilcoxon signed rank tests (superiority tests) were conducted to analyze the data in test and control drug of each case. It was also used for the data of total cases together. A *P* value of less than 0.05 was considered statistically significant for each test.

The trial protocol was approved by the Ethics Committee of Yueyang Hospital, Shanghai University of Traditional Chinese Medicine.

## 3. Results

### 3.1. The Results of the Preliminary Trial

The open-label preliminary trial was carried out according to the above evaluation criteria. With changes in patients self-rated symptom scores as the main outcomes, preliminary trials can obtain onset time after drug administration and efficacy maintenance time after drug withdrawal, so as to determine the observation period and the washout period.

15 patients with stable bronchiectasis (including three patients in this study) attended the open-label preliminary trial to observe the onset time point and the efficacy maintenance time point of the bronchiectasis stabilization decoction (control drug). After the administration of bronchiectasis stabilization decoction for two weeks, 5 out of 15 patients responded; the onset time ranged from 4 to 11 days; the washout periods ranged between 6 and 9 days. The rest 10 patients did not meet the onset standard during the two weeks of drug administration.

Three of the above 15 patients attended the preliminary trial to observe the onset time point and the efficacy maintenance time point of syndrome differentiation decoction (test drug). All of the three responded and they were numbered Case 1, Case 2, and Case 3.

The final results of the preliminary trial were as follows: Case 1 responded to syndrome differentiation decoction (test drug) but not to bronchiectasis stabilization decoction. The onset time was the 7th day and the washout period was 7 days. Case 2 responded to syndrome differentiation decoction (test drug) but not to bronchiectasis stabilization decoction. The onset time was the 10th day and the washout period was 8 days. Case 3 responded to both syndrome differentiation decoction (test drug) and bronchiectasis stabilization decoction; the onset times were the 11th day and 20th day, respectively; the washout periods were 9 days and 5 days, respectively.

Through the preliminary trial, the length of the observation periods in 3 circles for the three participants of N-of-1 RCTs was determined: both Case 1 and Case 2 took two weeks, and Case 3 took three weeks.

### 3.2. General Information of the Study

**Figure 2:**
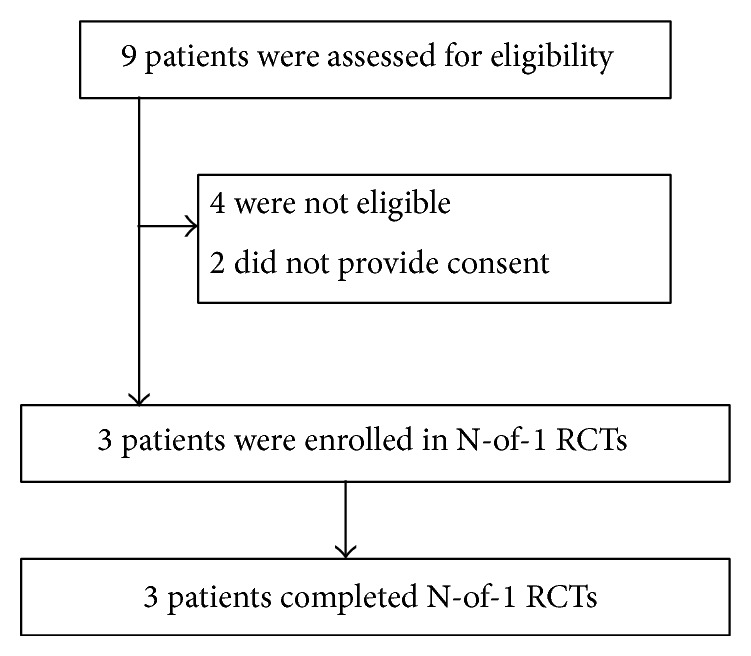
The flow chart of the whole process including recruitment, enrollment, and completion of the pilot study.

We conducted the study in the clinic of the Department of Respiratory Disease, Yueyang Hospital of Integrated Traditional Chinese and Western Medicine, Shanghai University of Traditional Chinese Medicine from October 2012 to June 2013. Five out of nine outpatients with stable bronchiectasis were eligible (recruitment rate 55.6%). Among the five patients meeting the inclusion criteria, three signed the informed consent form and were enrolled in this study (consent rate 60%) (Figure 2). The 3 patients enrolled included 1 male and 2 females, aged 57–74 years, and numbered as Case 1, Case 2, and Case 3. Based on the results of open-label preliminary trial, the observation period for Case 1, Case 2, and Case 3 was determined. All of the cases completed the single-patient, double-blind, randomized, multiple crossover design N-of-1 trial and the data analysis. Although all the three patients showed various degrees of improvement, no significant differences were found between individualized herbal decoction and control decoction on symptoms score, or on 24-hour sputum volume. However, one patient (Case 2) had clear preference for the individualized herbal decoction over the standard one with the confirmation after unblinding and was considered clinically important. Sections 3.3–3.5 showed the results in detail.

### 3.3. Case 1

*Medical History and Relevant Data*. Mr. Chu, 74 years old, developed cough, expectoration, small amount of thick yellow sputum, fatigue, poor sleep, dry mouth, spontaneous sweating, and joint pain; urine and stool were normal. (2012.5.15) Chest CT: old tuberculosis in the right upper lung, complicated by local bronchiectasis. The tongue was dark red and had cracks, the coating was thin and greasy, and the pulse was slippery. TCM syndrome differentiation was qi and yin deficiency with phlegm-heat.

*Treatment Rules*. To nourish yin, benefit qi, clear phlegm-heat, and tranquilize the mind.

*Syndrome Differentiation (Individualized) Decoction*. Radix Adenophorae 15 g, Radix Glehniae 15 g,* Astragalus membranaceus* 20 g, Rhizoma Polygonati 15 g, Rhizoma Fagopyri Dibotryis 30 g,* Scutellaria baicalensis *15 g, Herba Violae 20 g, Semen Coicis 20 g, Radix Asteris 10 g,* Salvia miltiorrhiza* 20 g,* Prunella vulgaris* 15 g,* Poria* with hostwood 10 g,* Angelica sinensis* 12 g, Cortex Albiziae 30 g, roasted Fructus Aurantii 12 g,* Polygala tenuifolia* 9 g, Rhizoma Acori Graminei 9 g,* Coptis chinensis* 6 g,* Geranium wilfordii* 15 g, Rhizoma Homalomenae 15 g, and licorice 5 g.

*Treatment Process*. The symptom score at the start of the trial (including the preliminary trial) was 21, and the 24 h sputum volume was 10 mL. During medication, the patient felt significant improvement in sleeping quality and relieved dry mouth and no gastrointestinal reactions; after the end of the second pair, the patient felt abdominal distention, back pain, cough, and yellow sputum; CRP of 27.3 mg/L was considered as acute exacerbation of bronchiectasis, so the patient received anti-infection treatment and discontinued the Chinese medicine. Three weeks after Chinese medicine withdrawal, the conditions were stable, and the patient started the third pair of the trial. During medication, the patient took the drug every day with no drugs left. The symptom score dropped 7 on a scale with sum score of 21. He did not take other drugs while taking the Chinese medicine, and the compliance was good. The patient felt improvement in the following symptoms: cough, expectoration, fatigue, spirit, sleep, and dry mouth, but joint pain. Tongue and pulse had no significant changes. The differences in symptom scores and 24 h sputum volume between the two decoctions were not statistically significant (Tables 1 and 2).

*Safety Outcome*. No obvious side effects occurred.

*Tendency to the Two Decoctions*. No tendency.

### 3.4. Case 2

*Medical History and Relevant Data*. Mrs. Wang, 64 years old, developed recurrent cough and yellow sputum for 40 years. Cough was frequent and sputum was yellow, thin, and in large volume, complicated by chest tightness, wheezing, spontaneous sweating, and poor sleep, but appetite was good, and the patient had a history of chronic gastritis and gastroesophageal reflux. Chest CT: bronchiectasis in lungs, complicated by infection; pulmonary bullous in the lungs. Tongue was light red, coating was thin and greasy, and pulse was thin and slippery. TCM syndrome differentiation was lung and spleen qi deficiency with phlegm-heat.

*Treatment Rules*. To tonify the spleen and benefit qi, clear the lung heat and reduce phlegm, and tranquilize the mind.

*Syndrome Differentiation (Individualized) Decoction*. Radix Ophiopogonis 15 g,* Poria cocos* 10 g,* Codonopsis pilosula* 10 g,* Astragalus membranaceus *15 g, Rhizoma Fagopyri Dibotryis 30 g,* Scutellaria baicalensis *20 g, Herba Violae 30 g,* Platycodon grandiflorum *5 g, Semen Coicis 30 g, Radix Asteris 10 g,* Inula* flower 9 g, calcined Concha Arcae 30 g, Pericarpium Citri Reticulatae Viride 6 g, cuttlebone 10 g,* Ardisia japonica* 30 g, Flos Farfarae 12 g,* Poria* with hostwood 20 g,* Angelica sinensis *10 g,* Prunella vulgaris *15 g, and Fructus Tritici Levis 15 g.

*Treatment Process*. The symptom score before the preliminary trial was 30, and the 24 h sputum volume was 60 mL. After medication, the patient felt that the symptoms were improved: cough and expectoration were improved slightly, and sleep was improved significantly. Her symptom score dropped from 30 to 23.57. On day 11 in the second pair, the patient took one diazepam tablet due to poor sleep, and after unblinding, it was known that the patient was then taking the bronchiectasis stabilization decoction (CD). Tongue and pulse had no significant changes. Although difference in overall symptom score between the two decoctions was not statistically significant (Table 1), the absolute difference of the mean symptom score between the two decoctions in two pairs of trial was ≥0.5 points, and in all of the three pairs, the patient could distinguish these two decoctions and medication sequence by their effects. After unblinding, it was known as the syndrome differentiation decoction. According to the standards of clinical efficacy, this decoction (ID) had clinical significance. Although the difference of 24 h sputum volume between the two decoctions was not statistically significant, the individualized decoction had the tendency to reduce more sputum volume (Table 2).

*Safety Outcome*. No obvious side effects occurred. 

*Tendency to the Two Decoctions*. Tendency to syndrome differentiation decoction (ID).

### 3.5. Case 3

*Medical History and Relevant Data*. Mrs. Wang, aged 57 years, had cough, large volume of yellow purulent sputum about 70 mL a day, with occasional wheezing, good appetite, good sleep, and normal urine and stool. In recent years, the patient visited many doctors, but the efficacy was not satisfactory. Before treatment, liver and kidney functions were normal. Chest CT: bronchiectasis in two lungs, complicated by infection and emphysema, and multiple bullae in the left lower lobe. The tongue was red, coating was yellow and greasy, and the pulse was slippery. TCM syndrome differentiation was diagnosed as phlegm-heat storing in lung accompanied by qi and yin insufficiency.

*Treatment Rule*. To clear the lung heat and reduce phlegm combined with nourishing qi and yin. 

*Syndrome Differentiation (Individualized) Decoction*.* Scutellaria baicalensis *30 g, Herba Violae 30 g, Rhizoma Fagopyri Cymosi 30 g,* Platycodon grandiflorum *10 g, Semen Coicis 30 g, Semen Benincasae 30 g,* Poria cocos* 15 g,* Astragalus membranaceus *15 g, Radix Asteris 15 g, Radix Adenophorae 15 g, Radix Ophiopogonis 15 g, liquorice 5 g,* Perilla* seed 15 g,* Magnolia officinalis* 10 g, Bryozoatum 30 g, and Concha Meretricis seu Cyclinae 30 g.

The drugs were planned to be used for three pairs, and each decoction was used for 3 weeks. However, the second period in the second pair only lasted 7 days because of an acute exacerbation of bronchiectasis. 

*Treatment Process*. Because the patient had severe bronchiectasis and had failed to respond to conventional treatment before the trial, high dose of some herbs in syndrome differentiation (individualized) decoction was given. The symptom score before the preliminary trial was 18, and the 24 h sputum volume was 70 mL. After the first pair, the patient felt that the conditions improved significantly and cough and expectoration were relieved. In the first period of the second pair, the conditions were stable; 7 days after the beginning of the second period in the second pair, the patient developed fever without any obvious causes, considered as acute exacerbation of bronchiectasis. Routine blood test (2013.3.4): WBC 15.0 × 10^9^/L, CRP 36.9 mg/L; liver and kidney functions were normal. Anti-infection treatment was provided, and the test drugs were discontinued. Seven weeks later, the patient returned to stable condition and the final pair was completed. During taking the Chinese medicine, no other drugs were used in combination, and the compliance was good. After unblinding, it was known that the patient developed acute exacerbation when taking the stabilization decoction. Tongue and pulse had no significant changes. After three pairs of treatment with the bronchiectasis stabilization decoction and the syndrome differentiation decoction, the patient felt that the treatment was quite successful. The symptom score dropped 4.57 scores on an 18 scale and the 24 h sputum volume reduced from 70 mL to 30 mL. However, the patient could not distinguish the two drugs, and the comparison in overall symptom score and 24 h sputum volume showed no statistically significant differences (Tables 1 and 2).

*Safety Outcome*. Although high dose of some herbs in syndrome differentiation (individualized) decoction were given, no obvious side effects occurred. The liver and kidney functions were normal. 

*Tendency to the Two Decoctions*. No tendency.

As there was an acute exacerbation of bronchiectasis during the second period of the second pair, the data of the second pair lost comparability. We therefore did not use the data of the second pair.

## 4. Discussion

### 4.1. Summary of the Results in this Study

It could be seen from the results of this study that N-of-1 RCTs were accepted and the patient actively cooperated. We found no significant differences between individualized herbal decoction and standard (control) decoction on symptoms score or on 24-hour sputum volume. Though Case 3 felt that her treatment was quite successful, she could not distinguish the two drugs. However, Case 2 had clear preference for the individualized herbal decoction over the standard one with the confirmation after unblinding and was considered clinically important according to standards of clinical criteria (Section 2.6).

No obvious side effects occurred during and after the 3 N-of-1 trials. We had proved the long term effects of syndrome differentiation treatment based on the bronchiectasis stabilization decoction in a previous randomized controlled trial [8]. The therapeutic principle of TCM in the treatment of bronchiectasis includes strengthening the body resistance, reducing phlegm, and clearing heat; its mechanisms of action have not been fully investigated.

Theoretically, the syndrome differentiation decoction should be superior to the control decoction. The lack of statistical significance between the two decoctions might be related to some factors, such as the rough estimation of drug washout period, relatively short observation period, and less pairs, indicating that the methods in this study could be further improved. N-of-1 trials must be constantly revised and improved before they are widely used in the field of TCM.

### 4.2. Comparing Findings with Other Studies

There have been a few articles of N-of-1 RCTs on the effect of traditional Chinese medicine [18–20]. These researches showed that N-of-1 RCTs were feasible and reflected the advantage of individualized treatment of TCM. However, the methodological qualities of these studies are questionable. One of the trials did not use blinding; no method for determining the washout period was reported in any of the trials.

Yuhong et al. reported a study of N-of-1 RCTs testing the effectiveness of Liuwei Dihuang decoction (LDD) for kidney-yin deficiency syndrome that is a traditional Chinese medicine syndrome in publicly clinical practice in China [21]. 47 patients completed 3 pairs of periods; only 3 (6.38%) were responders; among the whole group, neither the individual Likert score nor the SF-36 showed any statistical differences between LDD and placebo. The result of this study does not support the general application of LDD for patients with deficiency of kidney yin. The author speculated that a limitation of the trial was washout period which has not been fully considered, which resulted in residual effects of traditional Chinese medicine interfering with the differences between LDD and placebo. It was also suggested that more attention should be paid to choose experienced TCM doctor as investigator and keep the stimulant (placebo) the same with test medication in N-of-1 trial of TCM.

Compared with the above relevant studies, in this pilot study we tried to determine the washout period through preliminary trials, and we used two different Chinese medicine decoctions to make the randomized double-blind controlled trial. Bronchiectasis stabilization decoction used as the control was more easily accepted by the patients than placebo, especially in a trial which lasted for several months. Furthermore, the comparison between the control decoction (bronchiectasis stabilization decoction) and the individualized decoction may be the best way to reflect the individualized treatment of TCM.

### 4.3. The Feasibility of This Study

The result of this study showed that 60% of all eligible patients can be recruited; 100% of all recruited subjects completed the study and 100% of patients received every scheduled prescription of the study drug in a blinded manner; the clinical primary and secondary outcomes (patient self-rated symptom score on visual analogue scales and 24-hour sputum volume) were easily obtained and reliable. According to the criteria (Section 2.6.2(3)) for determining success of the pilot study, we concluded that this study was feasible with further modifications, for example, modifications of observation periods or pairs.

### 4.4. Strengths and Limitations

*(1) The Advantage of the Comparative Study of Two Different Chinese Medicine Decoctions*. Generally speaking, the existing traditional Chinese medicine has not been trialed by properly designed placebo-controlled trials, so it is difficult to find a reasonable and effective traditional Chinese medicine as positive control medicine in clinical trials [22]. Therefore, to verify the effectiveness of traditional Chinese medicine in N-of-1 RCTs, placebo control is the best choice. However, in the present Chinese cultural background, it is not easy persuading most of the subjects to accept even the placebo-controlled trials of TCM which are reasonably designed and fully comply with the ethical principles. In our study we had to adopt a form of compromise; bronchiectasis stabilization decoction which had been proved effective by RCT [8] was used as “positive control,” and test of superiority was used. Unlike the equivalence test, test drugs (individual prescription) could be evaluated as “effective” only if they were proved better than the control drug (bronchiectasis stabilization decoction).

Because of the unique perception, taste, and smell of traditional Chinese medicine, it is extremely difficult to find a control drug completely consistent with the test drug [23]. In our study the two TCM decoctions could be similar in appearance and size, but the difference in taste and smell may still exist. In order to compensate for this difference, we told the participants that both the test and control decoctions may be effective; the taste and smell did nothing with efficacy. This method seemed effective; all the three participants did not evaluate the efficacy by taste and smell of the decoctions. Two cases of three had no tendency to the decoctions. Thus we concluded that the implementation of overall blind method was generally successful.

As the use of decoction of Chinese medicinal herbs based on syndrome differentiation is still the mainstay of clinical practice, this form of clinical trials is closer to clinical practice. Clinicians and researchers should consider this kind of study design when conducting N-of-1 RCTs in TCM.

*(2) The Estimation of Washout Period of the Decoctions of TCM*. Since it is difficult to determine the process of Chinese medicine metabolism, Professor Gordon Guyatt suggested running a preliminary trial to determine the washout period along with the investigators' clinical experiences. Drug onset time and efficacy maintenance time after drug withdrawal were recorded from preliminary trials. The washout period was determined by these data. However, the estimation of the washout period might be questionable due to the validity of patients self-rated measurement method (severity of illness, symptom fluctuation, etc.). We think that the data obtained for preliminary trials can only be used as references. The estimation of washout period of the decoctions of TCM remains to be improved.

*(3) The Interference of Acute Exacerbation*. In this study, two cases developed acute exacerbation during the trial, resulting in trial discontinuation. Generally, in cases of acute exacerbation, we might discontinue N-of-1 RCT and provide conventional treatment. After conditions remitted and objective outcome measures returned to baseline levels, the next pair of trial could be restarted.

*(4) Statistic Analysis of N-of-1 Trial*. The statistical analysis of N-of-1 trial is challenging due to the autocorrelation generated from the repeated measure from single patient. We performed Wilcoxon signed rank test which took into account the repeated measure issue. However, the autocorrelation, time trends within each period are ignored due to the simplicity of the nonparametric test. It would be optimal to construct a proper statistical model that incorporates these features along with an estimation of treatment effect.

### 4.5. Implications for Clinical Practice and Future Research

*(1) The Importance of Setting Up Clinical Efficacy Criteria*. JAMA Users' Guide to Medical Literature stated [15] that the use of N-of-1 RCTs to improve patient care does not only depend on the statistical analysis of the results. For the three N-of-1 RCTs in this study, the statistical results showed no significant differences. However, the outcomes of Case 2 met the clinical efficacy criteria (Section 2.6) and therefore this case was considered as clinically important.

Lack of statistical significance may be partly related to less pairs (three pairs), together with other factors, such as the use of positive control drug, the rough estimation of washout period, and relatively short observation period. Our understanding is that the statistical analysis should be combined with clinical criteria for efficacy evaluation in the N-of-1 RCTs. The suggestion on setting up clinical efficacy standards proposed by Guyatt is necessary.

(*2) Exploring the Optimal Dose and Monitoring Drug Toxicity of Single Case*. There is a saying: “the secret of traditional Chinese medicine lies in the dosage.” In this study, Case 3 had severe bronchiectasis without responding to conventional western or TCM treatment before the trial. Based on the experience of distinguished veteran doctors of TCM, we increased the dose of* Scutellaria baicalensis* to 30 g in syndrome differentiation (individualized) decoction. This dose was three times higher than the upper limit value (9 g) of* Scutellaria baicalensis* in Chinese Pharmacopoeia. Under close monitoring of drug toxicity throughout the trial, Case 3 got significant improvement without gastrointestinal reaction or other adverse effects. Although we could not conclude that high dose of* Scutellaria baicalensis* was responsible for her improvement, it was proved safe for Case 3. There is still a big controversy on rational dosage of Chinese medicinal herbs [24]; thus some Chinese clinicians increased the dose of the herbs beyond the scope of Chinese Pharmacopoeia for the sake of getting better effects. That could be risky for both the clinicians and patients. N-of-1 RCTs may be more efficient and safer for exploring the optimal dose and monitoring drug toxicity of individuals.

## 5. Conclusion

This pilot study of N-of-1 RCTs compared the control decoction (bronchiectasis stabilization decoction, CD) with individualized decoctions (ID) in the treatment of 3 patients with stable bronchiectasis. Although we found no statistical significant differences between the two decoctions on all the outcomes, Case 2 had clear preference for ID over CD with the confirmation after unblinding and was considered clinically important according to clinical efficacy criteria. This form of clinical trials is closer to TCM clinical practice and had good compliance. It is necessary to set up clinical efficacy standards and pay attention to interference of acute exacerbation. While we are still not sure of the real value of N-of-1 RCTs in the research of individualized treatment of TCM before more cases are studied, the methods in this study were feasible and could be further improved.

## Supplementary Material

After three pairs of treatment with the bronchiectasis stabilization decoction and the syndrome differentiation decoction, the patient (case1) felt that the treatment was quite successful. However, the comparison in overall symptom score showed no statistically significant differences (Figure 1).
Although difference in overall symptom score between the two decoctions was not statistically significant (Figure 2), the absolute difference of the mean symptom score between the two decoctions in two pairs of trial ≥ 0.5 points.
The differences in overall symptom score of case 3 between the two decoctions were not statistically significant (Figure 3).
Table 1: All statistical analyses were performed using RStudio 0.98.953. One-sided paired Wilcoxon signed rank tests (superiority tests) were conducted to analyze the data in test and control drug of each case. It was also used for the data of total cases together. A *P*-value of less than 0.05 was considered statistically significant for each test. Although all the three patients showed various degree of improvement, no significant differences were found between individualized herbal decoction and control decoction on symptoms score, nor on 24 hours sputum volume.

## Figures and Tables

**Table 1:** Average symptom scores from the last week of each period and results of the statistical analyses.

	Case 1	Case 2	Case 3
Baseline	21	28	15
Pair1 CD	17.14	27.29	13
Pair1 ID	17	23.57	13
Pair2 ID	17.43	23.86	12.9
Pair2 CD	16.67	28.43	∗
Pair3 CD	14	26.33	12.38
Pair3 ID	14	27.57	13.43
*P* value^**^		0.417	
95% confidence interval^**^		(−1.05, *∝*)	

CD: control decoction; ID: individualized decoction.

^*^Not available due to an acute exacerbation.

^**^These values were the results of one-sided paired Wilcoxon signed rank test for all the 3 cases.

No statistically significant differences in average symptom score between the two decoctions for each of the 3 cases and for the total 3 cases together.

**Table 2:** Average 24 h sputum volume from the last week of each period and results of the statistical analyses.

	Case 1 (mL)	Case 2 (mL)	Case 3 (mL)
Baseline	10	62	40
Pair1 CD	5	60	22.5
Pair1 ID	5	53	38.6
Pair2 ID	7	55	30
Pair2 CD	5	65	∗
Pair3 CD	3	61	38.6
Pair3 ID	4	60	34.3
*P* value^**^		0.3674	
95% confidence interval^**^		(−7.55, *∝*)	

CD: control decoction; ID: individualized decoction.

^*^Not available due to an acute exacerbation.

^**^These values were the results of one-sided paired Wilcoxon signed rank test for all the 3 cases.

No statistically significant differences in average 24 h sputum volume between the two decoctions for each of the 3 cases and for the total 3 cases together.
