# Supplementary material for: Evaluating the Individualized Treatment of Traditional Chinese Medicine: A Pilot Study of N-of-1 Trials
Source: Evid Based Complement Alternat Med. 2014 Nov 11;2014:148730. doi: 10.1155/2014/148730 (PMC4244929; doi:10.1155/2014/148730)
Supplement: Supplementary file 1 — After three pairs of treatment with the bronchiectasis stabilization decoction and the syndrome differentiation decoction, the patient (case1) felt that the treatment was quite successful. However, the comparison in overall symptom score showed no statistically significant differences (Figure 1). Although difference in overall symptom score between the two decoctions was not statistically significant (Figure 2), the absolute difference of the mean symptom score between the two decoctions in two pairs of trial ≥ 0.5 points. The differences in overall symptom score of case 3 between the two decoctions were not statistically significant (Figure 3). Table 1: All statistical analyses were performed using RStudio 0.98.953. One-sided paired Wilcoxon signed rank tests (superiority tests) were conducted to analyze the data in test and control drug of each case. It was also used for the data of total cases together. A P-value of less than 0.05 was considered statistically significant for each test. Although all the three patients showed various degree of improvement, no significant differences were found between individualized herbal decoction and control decoction on symptoms score, nor on 24 hours sputum volume. [file 148730.f1.pdf]

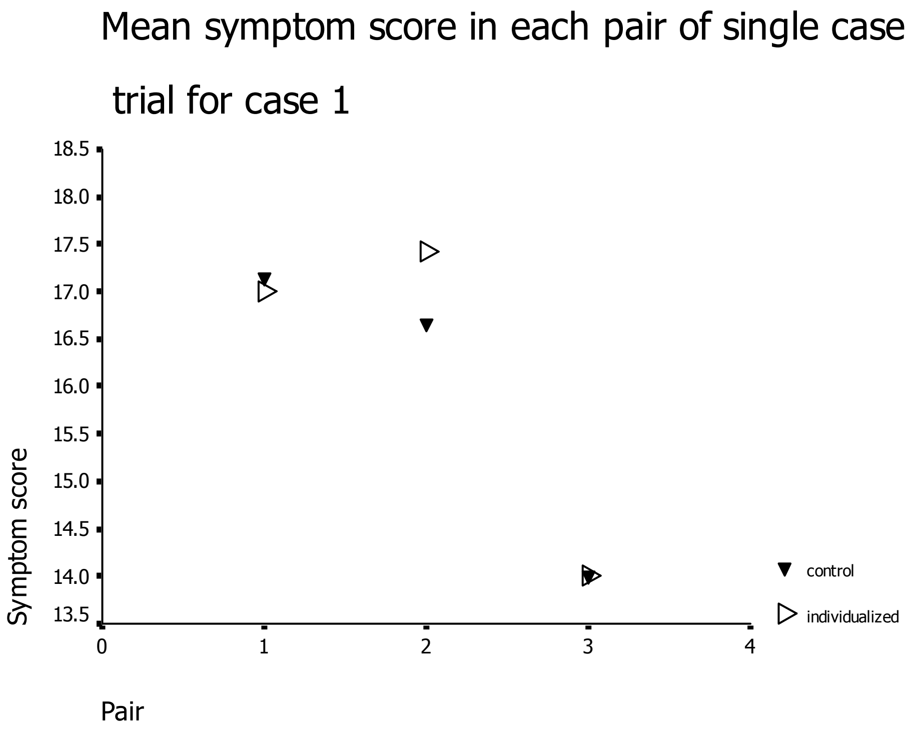

Figure 1: Mean symptom score in each pair of single case trial for case 1

After three pairs of treatment with the bronchiectasis stabilization decoction and the syndrome differentiation decoction, the patient (case1) felt that the treatment was quite successful. However, the comparison in overall symptom score showed no statistically significant differences (Figure 1).

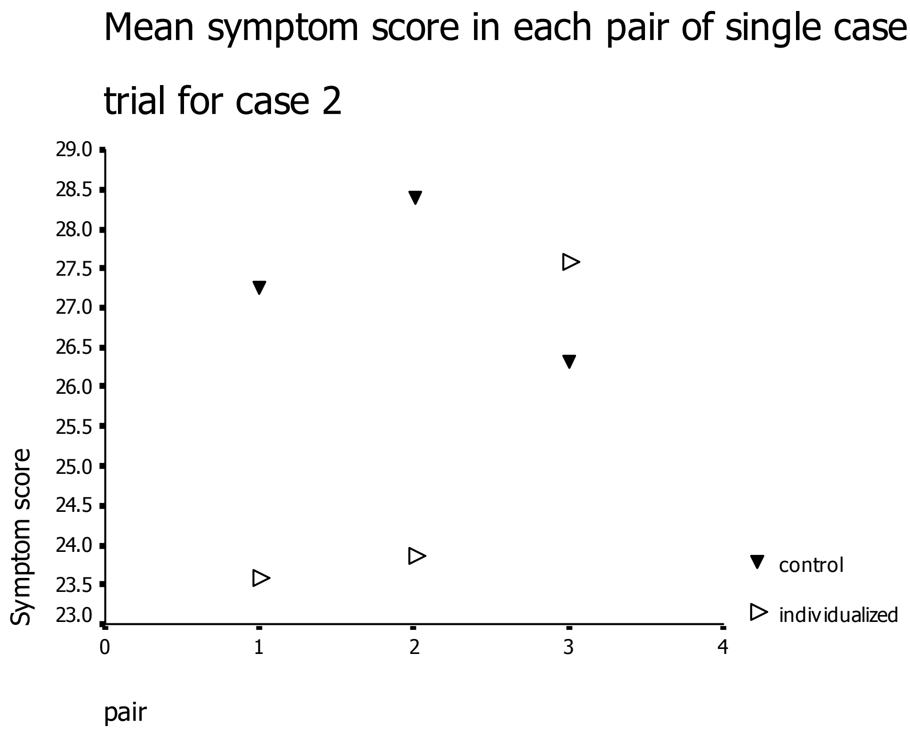

Figure 2: Mean symptom score in each pair of single case trial for case 2

Although difference in overall symptom score between the two decoctions was not statistically significant(Figure 2), the absolute difference of the mean symptom score between the two decoctions in two pairs of trial ≥ 0.5 points.

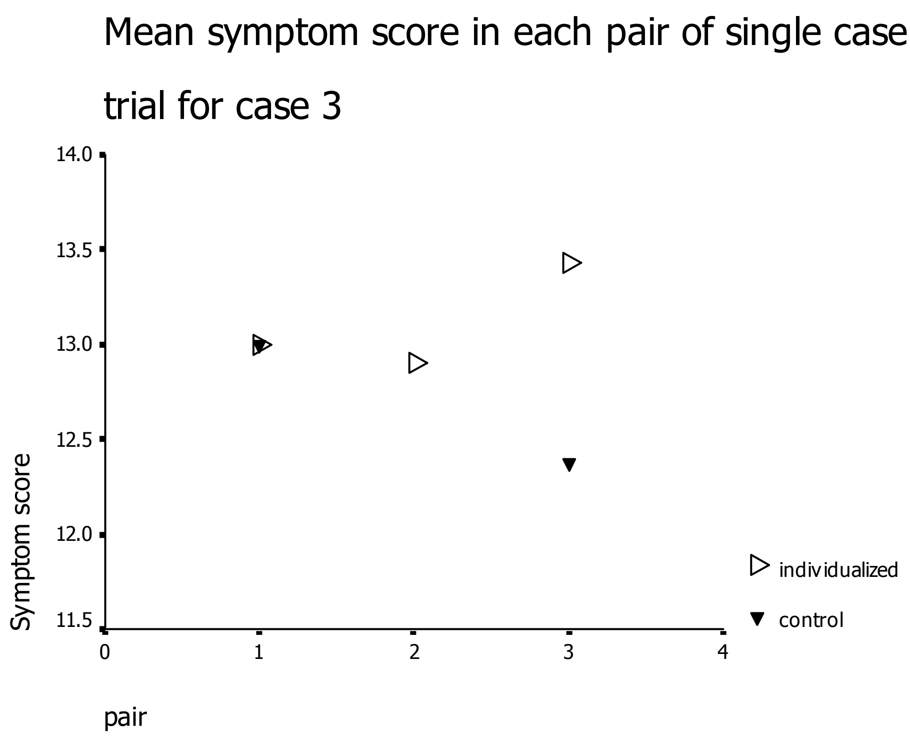

Figure 3: Mean symptom score in each pair of single case trial for case 3

The differences in overall symptom score of case 3 between the two decoctions were not statistically significant(Figure 3).

Table 1. Mean symptom score in the last week of each period and results of paired t test of all the 3 cases.

	Case 1	Case 2	Case 3
Baseline	21	30	18
Pair1 CP	17.14	27.29	13
Pair1 IP	17	23.57	13
Pair2 IP	17.43	23.86	12.9
Pair2 CP	16.67	28.43	*
Pair3 CP	14	26.33	12.38
Pair3 IP	14	27.57	13.43
Mean Difference	0.21	-2.25	-0.525
SD	0.48	3.08	0.74
t score	0.74	-1.30	-1.0
P-value	0.54	0.32	0.5
95% Confidence Interval	-1.00, 1.41	-10.15, 5.45	-7.2, 6.15

CP: control decoction; IP: individualized decoction.

* not available due to an acute exacerbation.

No statistically significant differences in overall symptom score between the two decoctions for all the 3 cases.
